# Preoperative Pan-Immune-Inflammation Value for the Differential Diagnosis and Prognostic Assessment of Ovarian Tumors: A Retrospective Cohort Study

**DOI:** 10.3390/diagnostics16121873

**Published:** 2026-06-16

**Authors:** Süleyman Özen, Muzaffer Sancı

**Affiliations:** Department of Gynecologic Oncology, İzmir City Hospital, İzmir 35540, Turkey; drsanci@yahoo.com

**Keywords:** ovarian neoplasms, pan-immune-inflammation value, CA-125, biomarkers, systemic inflammation, differential diagnosis, borderline ovarian tumor, prognosis

## Abstract

**Background/Objectives**: Preoperative differentiation of benign, borderline, and malignant ovarian tumors remains challenging. The pan-immune-inflammation value (PIV), a composite inflammation-based biomarker derived from routine blood counts, may contribute to ovarian tumor characterization. This study aimed to evaluate the diagnostic and prognostic significance of PIV in ovarian tumors. **Methods**: This retrospective single-center cohort study included 316 patients with histopathologically confirmed ovarian tumors classified as benign (*n* = 139), borderline (*n* = 61), or malignant (*n* = 116). Preoperative inflammatory indices were calculated from peripheral blood samples obtained within 24 h before surgery. Diagnostic performance was assessed using receiver operating characteristic (ROC) curve analysis and multivariable logistic regression. Progression-free survival (PFS) and overall survival (OS) were evaluated in malignant cases with available follow-up. **Results**: Inflammatory indices increased significantly from benign to borderline and malignant tumors (*p* < 0.001 for all). Among inflammatory markers, PIV demonstrated the highest diagnostic performance, with an area under the curve (AUC) of 0.852 (95% CI 0.808–0.893), sensitivity of 75.0%, and specificity of 80.5%. CA-125 demonstrated the highest overall diagnostic accuracy (AUC 0.978, 95% CI 0.961–0.991). Pairwise ROC analyses showed consistent discriminatory performance of PIV across ovarian tumor comparisons. In multivariable analysis, age, tumor size, ln(CA-125), and ln(PIV) were independently associated with malignancy (all *p* < 0.05). The combined CA-125 + PIV model demonstrated a statistically significant but modest improvement in diagnostic performance compared with CA-125 alone. During a median follow-up of 26.9 months (IQR 22.2–32.5), PIV was not significantly associated with PFS or OS. **Conclusions**: PIV demonstrated the highest diagnostic performance among evaluated inflammatory indices and may provide complementary information for preoperative epithelial ovarian tumor assessment. However, CA-125 remained the most accurate standalone biomarker, and the prognostic value of PIV appeared limited.

## 1. Introduction

Ovarian cancer remains one of the most lethal gynecologic malignancies and continues to pose a significant global health burden. According to recent global cancer statistics, approximately 20 million new cancer cases and nearly 10 million cancer-related deaths were recorded worldwide in 2022, underscoring the magnitude of cancer as a leading cause of morbidity and mortality [1]. Ovarian cancer alone accounts for more than 320,000 newly diagnosed cases and over 200,000 deaths annually, reflecting its disproportionate contribution to cancer-related mortality relative to its incidence [2].

The preoperative distinction between benign, borderline, and malignant ovarian tumors remains challenging in routine clinical practice. Borderline ovarian tumors are particularly problematic because they represent an intermediate group with distinct biological behavior and management implications, yet often overlap with both benign and malignant lesions in terms of imaging and biomarker profiles [3]. Current diagnostic assessment relies on imaging findings together with serum biomarkers, most notably cancer antigen 125 (CA-125). However, CA-125 has limited specificity, especially in premenopausal women and in several benign gynecologic or inflammatory conditions, which reduces its value as a stand-alone marker [3,4]. These limitations underscore the need for additional, inexpensive, and readily available biomarkers that may improve preoperative risk stratification.

Systemic inflammation has increasingly been recognized as a major component of cancer development and progression [5]. Chronic inflammatory signaling contributes to tumor initiation, proliferation, angiogenesis, invasion, and immune escape, while the interaction between tumor cells and the host immune system shapes disease behavior and clinical outcomes [6]. In ovarian cancer, inflammation-based indices derived from routine complete blood count parameters have attracted growing interest as accessible biomarkers reflecting the balance between protumor inflammation and antitumor immunity. Among these, the neutrophil-to-lymphocyte ratio (NLR), platelet-to-lymphocyte ratio (PLR), and monocyte-to-lymphocyte ratio (MLR) have all been investigated as potential diagnostic and prognostic markers [7,8]. Platelets are of particular interest because they are not passive bystanders, but active mediators of tumor progression and metastatic dissemination, providing a biological rationale for platelet-containing inflammatory scores [9].

More recently, the pan-immune-inflammation value (PIV), which integrates neutrophil, platelet, monocyte, and lymphocyte counts into a single composite parameter, has been proposed as a novel biomarker reflecting systemic immune–inflammatory status [10]. By capturing multiple components of the immune response, PIV provides a more comprehensive assessment compared with conventional indices. Emerging evidence suggests that PIV may be associated with oncologic outcomes and disease burden; however, its clinical role in ovarian tumors remains insufficiently defined, particularly in studies including benign and borderline lesions alongside malignant cases [10,11].

Furthermore, the prognostic value of PIV may vary according to patient selection and treatment context. Patients undergoing complete cytoreductive surgery represent a relatively homogeneous cohort, allowing for a more precise evaluation of the relationship between systemic inflammation and survival outcomes. Nevertheless, the prognostic significance of PIV in such well-defined populations, especially in the presence of standardized treatment and follow-up, remains to be clarified.

In this context, the present study aimed to evaluate the diagnostic performance of the pan-immune-inflammation value in differentiating benign, borderline, and malignant ovarian tumors and to investigate its prognostic significance in patients with malignant ovarian tumors who underwent complete cytoreductive surgery and had available follow-up data.

## 2. Materials and Methods

This retrospective single-center cohort study included patients with histopathologically confirmed ovarian tumors who underwent primary surgery either at our institution or at external referral centers and subsequently received postoperative follow-up at the Gynecologic Oncology Department of İzmir City Hospital.

Patients were screened during the data-retrieval period between December 2023 and January 2026. For patients initially operated at external referral centers, the date of primary surgery could precede the institutional follow-up review period; operative and histopathological records were retrieved retrospectively through the national electronic medical record system.

A total of 316 consecutive patients with histopathologically confirmed ovarian tumors were enrolled and categorized into three groups: benign (*n* = 139), borderline (*n* = 61), and malignant (*n* = 116).

Patients were eligible for inclusion if they underwent surgical treatment for an ovarian mass, had complete preoperative laboratory data, available clinicopathological records, and documented follow-up data. In the malignant cohort, only patients who underwent complete cytoreductive surgery with no residual tumor (R0 resection) were included. Patients were excluded if they had active infection or inflammatory disease, hematological or autoimmune disorders, were receiving immunosuppressive therapy, had incomplete data, had received preoperative blood transfusion, or had undergone neoadjuvant chemotherapy prior to surgery. Potential infectious or inflammatory conditions were identified retrospectively through review of clinical records, laboratory findings, medication history, discharge summaries, and physician documentation available in the institutional electronic medical record system. There were no missing data among the variables included in the final analyses, as patients with incomplete laboratory or clinicopathological records were excluded during cohort assembly.

Demographic, clinical, and laboratory data were retrospectively obtained from electronic medical records. Collected variables included age, body mass index (BMI), parity, smoking status, tumor size, serum CA-125 levels, FIGO stage, and histopathological subtype. Preoperative laboratory parameters were obtained from peripheral venous blood samples collected within 24 h before surgery to ensure temporal consistency between laboratory assessment and surgical intervention. Blood samples were obtained before surgery and prior to administration of perioperative antibiotics or other major therapeutic interventions that could potentially influence systemic inflammatory parameters. Serum CA-125 levels were measured using the Roche cobas platform (Roche Diagnostics, Basel, Switzerland), whereas complete blood count parameters were analyzed using the Sysmex XN-3000 (Sysmex Corporation, Kobe, Japan) hematology analyzer according to institutional laboratory protocols. For patients referred from external centers, complete confirmation of the analytical platforms used for all preoperative laboratory measurements was not available. PIV was calculated from complete blood count parameters.

Neutrophil-to-lymphocyte ratio (NLR), platelet-to-lymphocyte ratio (PLR), and monocyte-to-lymphocyte ratio (MLR) were calculated as ratios of respective cell counts. The pan-immune-inflammation value (PIV) was calculated as neutrophil × platelet × monocyte divided by lymphocyte. All hematological parameters were expressed in 10^9^/L.

For prognostic analysis, only patients in the malignant group with available follow-up data were included. Follow-up duration was calculated from the date of primary surgery to the date of last clinical follow-up or death. Progression-free survival (PFS) was defined as the time from surgery to disease recurrence, progression, or death, whichever occurred first. Overall survival (OS) was defined as the time from surgery to death from any cause. Patients without events were censored at the date of last follow-up.

All statistical analyses were conducted using IBM SPSS Statistics for Windows, version 28.0 (IBM Corp., Armonk, NY, USA). Continuous variables were expressed as median and interquartile range (IQR), and categorical variables as frequency and percentage. Normality of distribution was assessed using the Shapiro–Wilk test, and non-parametric tests were applied accordingly.

Comparisons among benign, borderline, and malignant groups were performed using the Kruskal–Wallis test, followed by Bonferroni-corrected post hoc analyses. Categorical variables were compared using the chi-square or Fisher’s exact test. Additional pairwise ROC analyses were performed to evaluate the discriminatory performance of inflammatory indices between benign and borderline tumors, borderline and malignant tumors, and benign and malignant tumors separately.

Receiver operating characteristic (ROC) curve analysis was used to evaluate diagnostic performance, and area under the curve (AUC), 95% confidence intervals, sensitivity, specificity, and optimal cut-off values determined by the Youden index were calculated. Comparisons between correlated ROC curves, including CA-125 alone versus the combined CA-125 + PIV model, were additionally performed using the DeLong method.

For prognostic evaluation, patients in the malignant cohort were stratified into low- and high-PIV groups according to the median PIV. Kaplan–Meier survival analysis was used to estimate PFS and OS, and survival curves were compared using the log-rank test.

The proportional hazards assumption was evaluated before Cox regression analysis using log-minus-log survival plots, Schoenfeld residual-based assessment, and time-interaction diagnostics.

Univariate and multivariable Cox proportional hazards regression analyses were performed to identify factors associated with PFS and OS. For diagnostic multivariable logistic regression, age, tumor size, ln(CA-125), and ln(PIV) were entered as clinically relevant prespecified variables, and results were reported as odds ratios with 95% confidence intervals. Multicollinearity among predictors included in the multivariable logistic regression model was assessed using variance inflation factors (VIFs). Model calibration was evaluated using the Hosmer–Lemeshow goodness-of-fit test. Internal validation was additionally performed using 10-fold cross-validation, in which the prespecified logistic regression model was refitted within each fold using the same predefined variables. No data-driven variable reselection was performed before cross-validation. Model discrimination was assessed by calculating the mean area under the curve (AUC) across validation folds.

Spearman correlation analysis was used to evaluate relationships between PIV and clinicopathological variables. A *p*-value < 0.05 was considered statistically significant.

No a priori sample size calculation was performed because this was a retrospective study based on a consecutive cohort of eligible patients treated during the study period.

This retrospective study was conducted in accordance with the Declaration of Helsinki and was approved by the Ethics Committee of İzmir City Hospital (dated 11 March 2026, decision no: 226/163). Due to the retrospective nature of the study, the requirement for informed consent was waived.

## 3. Results

**Figure 1:**
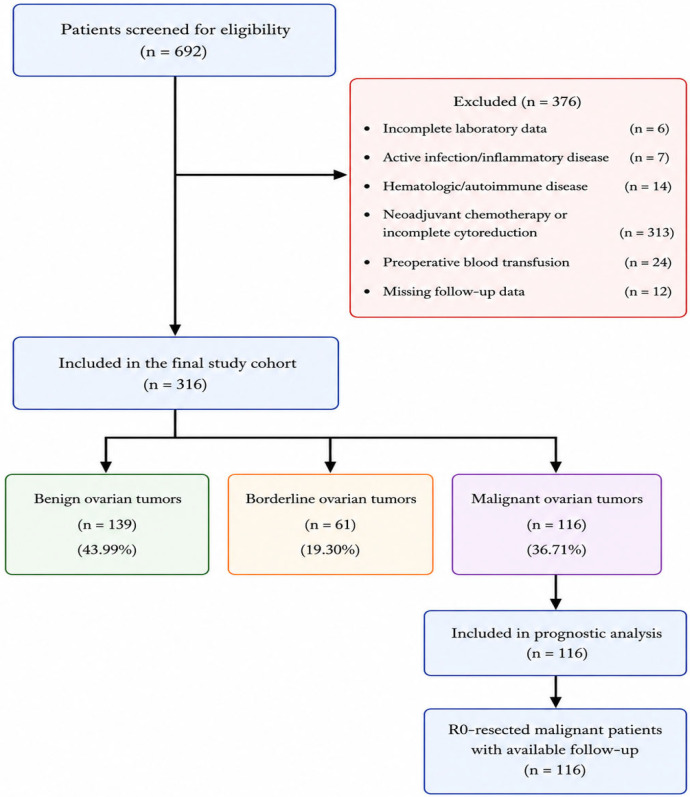
STROBE-style flow diagram of patient selection, exclusion criteria, cohort allocation, and inclusion in prognostic analyses.

Patient selection, exclusion criteria, cohort allocation, and inclusion in prognostic analyses are summarized in Figure 1. A total of 692 patients were screened for eligibility, of whom 376 were excluded according to predefined criteria. The final study cohort consisted of 316 patients, including 139 benign, 61 borderline, and 116 malignant ovarian tumors.

A total of 316 patients were included, comprising 139 benign, 61 borderline, and 116 malignant ovarian tumors. Baseline clinicopathological characteristics are presented in Table 1. Age differed significantly across groups (Kruskal–Wallis test, *p* < 0.001), with median values of 60.0 years (IQR 49.5–68.0) in benign tumors, 44.0 years (IQR 32.0–50.0) in borderline tumors, and 59.5 years (IQR 50.0–68.0) in malignant tumors. Body mass index also differed significantly (*p* = 0.001), with median values of 28.0 (IQR 25.6–31.0), 27.1 (IQR 24.9–28.8), and 26.1 (IQR 23.9–28.4), respectively. Tumor size increased across groups (median 5.80 cm vs. 8.20 cm vs. 9.05 cm, *p* < 0.001). Serum CA-125 levels were 18.90 U/mL (IQR 14.45–25.55), 43.80 U/mL (IQR 31.70–55.60), and 182.30 U/mL (IQR 103.88–307.48), respectively (*p* < 0.001). Among borderline tumors, 78.7% were stage I–II, whereas 73.3% of malignant tumors were stage III–IV.

PIV differed significantly among groups (Kruskal–Wallis test, *p* < 0.001 for all variables), as shown in Table 2. Median PIVs were 264.13 (IQR 193.21–343.11) in benign tumors, 399.39 (IQR 313.32–486.38) in borderline tumors, and 524.28 (IQR 408.62–711.46) in malignant tumors. Post hoc pairwise comparisons with Bonferroni correction demonstrated significant differences between all groups (all *p* < 0.001). Median NLR values were 2.26 (IQR 1.77–2.73), 2.48 (IQR 2.22–2.84), and 3.05 (IQR 2.56–3.96), respectively (*p* < 0.001). Median PLR values were 129.44 (IQR 110.89–151.68), 154.32 (IQR 137.74–179.76), and 190.91 (IQR 154.80–255.32), respectively (*p* < 0.001). Median MLR values were 0.21 (IQR 0.17–0.24), 0.24 (IQR 0.20–0.27), and 0.29 (IQR 0.22–0.36), respectively (*p* < 0.001).

**Figure 2:**
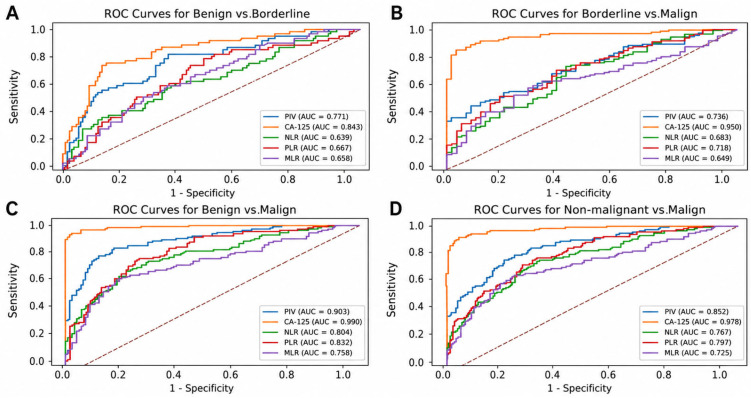
Pairwise receiver operating characteristic (ROC) curve analyses for the differential diagnosis of ovarian tumors. Panel (**A**) shows the comparison between benign and borderline tumors, panel (**B**) between borderline and malignant tumors, panel (**C**) between benign and malignant tumors, and panel (**D**) between non-malignant and malignant tumors. In all pairwise comparisons, CA-125 demonstrated the highest diagnostic accuracy. PIV showed consistent discriminatory ability across all analyses, with stronger performance in comparisons involving malignant tumors than in the borderline versus malignant setting.

The diagnostic performance of inflammatory markers and CA-125 was evaluated using receiver operating characteristic (ROC) curve analyses across different ovarian tumor comparisons, as shown in Figure 2. In the overall non-malignant versus malignant analysis, PIV demonstrated the highest diagnostic performance among inflammatory indices, with an area under the curve (AUC) of 0.852 (95% CI 0.808–0.893), sensitivity of 75.0%, and specificity of 80.5% at an optimal cut-off value of 410.97. The corresponding AUC values for NLR, PLR, and MLR were 0.767, 0.797, and 0.725, respectively. CA-125 demonstrated the highest overall diagnostic accuracy, with an AUC of 0.978 (95% CI 0.961–0.991), sensitivity of 91.4%, and specificity of 96.0% at a cut-off value of 66.95 U/mL.

Additional pairwise ROC analyses were performed for benign versus borderline, borderline versus malignant, benign versus malignant, and non-malignant versus malignant tumor comparisons (Figure 2). In the benign versus borderline comparison, PIV demonstrated an AUC of 0.771, whereas CA-125 yielded an AUC of 0.843. In the borderline versus malignant comparison, the AUC values for PIV and CA-125 were 0.736 and 0.950, respectively. The highest discriminatory performance of PIV was observed in comparisons involving benign and malignant tumors directly, with AUC values of 0.903 for benign versus malignant discrimination and 0.852 for non-malignant versus malignant discrimination.

Overall, PIV demonstrated consistent discriminatory performance across different ovarian tumor comparisons, although its diagnostic accuracy remained lower than that of CA-125 in all analyses.

**Figure 3:**
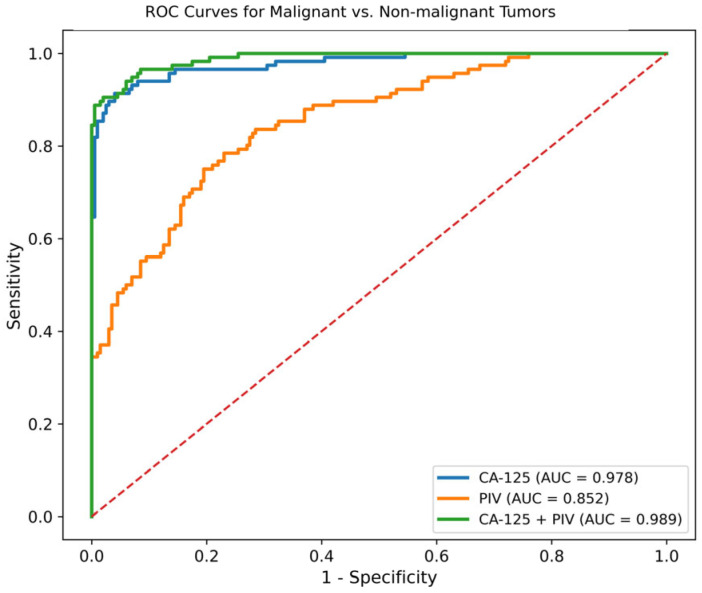
Receiver operating characteristic (ROC) curves of CA-125 alone, PIV alone, and the combined CA-125 + PIV model for discrimination of malignant versus non-malignant ovarian tumors. The combined model demonstrated the highest overall diagnostic performance.

Figure 3 shows the additional ROC analysis to evaluate the potential incremental diagnostic contribution of PIV beyond CA-125 in distinguishing malignant from non-malignant ovarian tumors. CA-125 alone demonstrated an AUC of 0.978, whereas PIV alone yielded an AUC of 0.852. A combined model incorporating both CA-125 and PIV achieved an AUC of 0.989, indicating a modest improvement in discriminatory performance compared with CA-125 alone. DeLong comparison analysis demonstrated that the combined CA-125 + PIV model yielded a significantly higher AUC than CA-125 alone (0.989 vs. 0.978, *p* = 0.013).

Multivariable logistic regression analysis is presented in Table 3. Age (OR 1.10, 95% CI 1.04–1.17, *p* = 0.001), tumor size (OR 1.65, 95% CI 1.18–2.32, *p* = 0.004), ln(CA-125) (OR 136.96, 95% CI 25.02–749.76, *p* < 0.001), and ln(PIV) (OR 63.81, 95% CI 7.13–571.21, *p* < 0.001) were independently associated with malignancy.

Variance inflation factor analysis demonstrated no evidence of prohibitive multicollinearity among age, tumor size, ln(CA-125), and ln(PIV), with all VIF values below 2. The multivariable logistic regression model showed acceptable calibration according to the Hosmer–Lemeshow goodness-of-fit test (χ^2^ = 2.90, df = 8, *p* = 0.940). Internal validation using 10-fold cross-validation yielded a mean AUC of 0.993 (SD 0.011), supporting the stability and discriminatory performance of the model.

Spearman correlation analysis demonstrated a correlation between PIV and CA-125 (rho = 0.546, *p* < 0.001) and between PIV and tumor size (rho = 0.402, *p* < 0.001).

Detailed clinicopathological characteristics of the malignant cohort according to preoperative PIV groups are summarized in Table 4. No significant differences were observed between low- and high-PIV groups regarding age, tumor size, CA-125 levels, histologic subtype distribution, FIGO stage, progression/recurrence status, or death rates (all *p* > 0.05), whereas PIVs differed significantly by definition (*p* < 0.001).

Prognostic analysis was performed in the malignant cohort (*n* = 116). The median follow-up duration in the malignant cohort was 26.9 months (IQR 22.2–32.5), with a maximum follow-up of 36.0 months. During follow-up, 46 patients (39.7%) experienced progression or recurrence and 23 patients (19.8%) died. Patients were stratified into low- and high-PIV groups using the malignant-group median PIV of 524.28, yielding 58 patients in each group.

**Figure 4:**
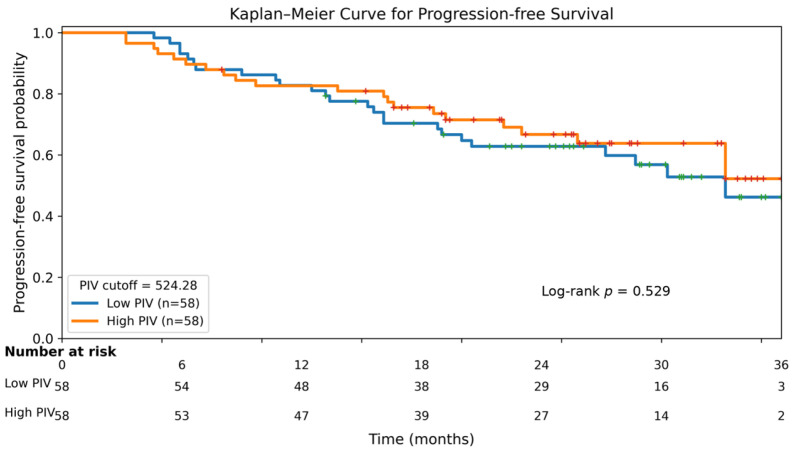
Kaplan–Meier curves for PFS according to preoperative PIV groups in the malignant ovarian tumor cohort. Patients were stratified into low- and high-PIV groups using the malignant-group median PIV of 524.28, yielding 58 patients in each group. Plus (+) symbols indicate censored observations. No statistically significant difference in PFS was observed between the groups (log-rank *p* = 0.529). Numbers at risk are provided below the *x*-axis.

Kaplan–Meier analysis for PFS is shown in Figure 4. Median PFS was 33.2 months in the low-PIV group and was not reached in the high-PIV group. The estimated 3-year PFS rates were 46.2% in the low-PIV group and 52.2% in the high-PIV group. No statistically significant difference was observed between groups (log-rank *p* = 0.529).

**Figure 5:**
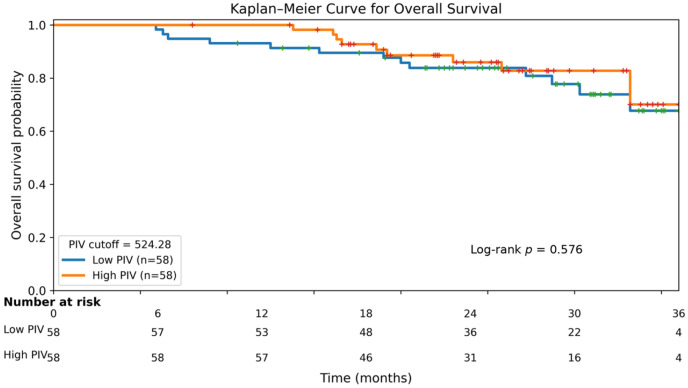
Kaplan–Meier curves for OS according to preoperative PIV groups in the malignant ovarian tumor cohort. Patients were stratified into low- and high-PIV groups using the malignant-group median PIV of 524.28, yielding 58 patients in each group. Plus (+) symbols indicate censored observations. No statistically significant difference in OS was observed between the groups (log-rank *p* = 0.576). Numbers at risk are provided below the *x*-axis.

Kaplan–Meier analysis for OS is presented in Figure 5. Median overall survival was not reached in either group. The estimated 3-year OS rates were 67.7% in the low-PIV group and 70.1% in the high-PIV group. No statistically significant difference was observed between groups (log-rank *p* = 0.576).

Cox proportional hazards regression analysis is presented in Table 5. Preoperative PIV was not significantly associated with either PFS or OS when analyzed as a dichotomized variable or as a ln-transformed continuous parameter. In univariable analysis, high versus low PIV was not associated with PFS (HR 0.83, 95% CI 0.46–1.48, *p* = 0.531) or OS (HR 0.79, 95% CI 0.35–1.81, *p* = 0.579). Likewise, ln(PIV) showed no significant association with PFS (HR 0.93, 95% CI 0.44–1.93, *p* = 0.835) or OS (HR 1.60, 95% CI 0.55–4.62, *p* = 0.389). In multivariable models adjusted for age, FIGO stage, tumor size, and ln(CA-125), PIV remained non-significant for both survival endpoints. None of the variables included in the PFS model reached statistical significance, whereas ln(CA-125) showed a borderline association with OS that did not reach the conventional threshold for significance (HR 1.83, 95% CI 0.90–3.69, *p* = 0.093). Overall, these findings indicate that preoperative PIV did not provide independent prognostic information for either progression-free or overall survival in the malignant cohort.

The proportional hazards assumption was assessed before Cox regression analysis. No apparent violation was observed for PIV in either the PFS model (*p* = 0.288) or the OS model (*p* = 0.600). In contrast, some clinicopathological covariates showed evidence of potential time-dependent effects, including FIGO stage (*p* = 0.019) and ln(CA-125) (*p* = 0.048) in the PFS model, and age (*p* = 0.001), tumor size (*p* = 0.008), and ln(CA-125) (*p* = 0.005) in the OS model.

## 4. Discussion

In the present study, preoperative PIV, NLR, PLR, and MLR increased progressively from benign to borderline and malignant ovarian tumors, indicating that systemic inflammatory burden parallels tumor aggressiveness. Among the evaluated inflammatory markers, PIV showed the best diagnostic performance (AUC 0.852, 95% CI 0.808–0.893) and remained independently associated with malignancy, whereas CA-125 retained the highest overall discriminatory accuracy (AUC 0.978, 95% CI 0.961–0.991). In contrast, PIV was not significantly associated with PFS or OS in the malignant cohort (log-rank *p* = 0.529 and *p* = 0.576, respectively).

These findings are biologically plausible, as tumor-associated inflammation contributes to angiogenesis, invasion, metastatic spread, and immune escape [12,13]. Previous studies have similarly shown that inflammatory indices are elevated in malignant ovarian tumors compared with benign lesions [7,14,15]. In particular, combined inflammation-based models have demonstrated improved diagnostic performance over individual markers [14]. Within this framework, the relatively stronger performance of PIV among inflammation-based indices in our cohort may reflect its more comprehensive integration of protumor inflammatory and host immune components.

A notable strength of the present study is the separate evaluation of borderline ovarian tumors, which remain one of the most challenging areas of preoperative ovarian tumor assessment because conventional serum markers often demonstrate limited specificity in this intermediate biological setting. Satır Özel et al. reported that preoperative tumor marker elevations in borderline ovarian tumors were not consistently associated with histopathological subtype [16]. Similarly, Ronsini et al. demonstrated that combining inflammatory indices with CA-125 improved discrimination between borderline and malignant ovarian tumors [17]. Consistent with these findings, Huang et al. showed that combined use of CA-125, neutrophil-to-lymphocyte ratio, and platelet-to-lymphocyte ratio improved the diagnostic differentiation of borderline and malignant epithelial ovarian tumors [18]. In addition, Yun et al. reported that both neutrophil-to-lymphocyte and platelet-to-lymphocyte ratios differed significantly across benign, borderline, and malignant ovarian tumors, supporting the role of systemic inflammatory markers in preoperative ovarian tumor stratification [19]. In the present study, additional pairwise ROC analyses demonstrated a progressive increase in inflammatory burden across benign, borderline, and malignant ovarian tumors. PIV showed moderate discriminatory performance in benign-versus-borderline (AUC 0.771) and borderline-versus-malignant (AUC 0.736) comparisons, whereas substantially stronger performance was observed for benign-versus-malignant discrimination (AUC 0.903). These findings suggest that systemic inflammatory activation becomes more pronounced with increasing malignant potential, while the relatively lower discriminatory performance observed in borderline-related comparisons may reflect the intermediate biological behavior and overlapping inflammatory characteristics of borderline ovarian tumors.

Despite the strong diagnostic performance of PIV (AUC 0.852, 95% CI 0.808–0.893), CA-125 remained the most accurate single marker for malignancy (AUC 0.978, 95% CI 0.961–0.991). Although CA-125 retained the highest standalone diagnostic accuracy, the combined CA-125 + PIV model demonstrated a modest improvement in overall discriminatory performance. DeLong comparison analysis additionally demonstrated that the combined CA-125 + PIV model achieved a statistically significant but relatively modest improvement in discriminatory performance compared with CA-125 alone. This finding suggests that inflammation-based biomarkers may provide complementary biological information beyond conventional tumor markers alone, although the magnitude of incremental benefit appears limited. These findings are consistent with previous studies indicating that inflammatory indices may be most informative when integrated with established tumor markers or multimarker prediction models [14,20,21]. In particular, previous studies have shown that combining CA-125 with inflammatory indices such as NLR and PLR may improve preoperative discrimination of borderline and malignant epithelial ovarian tumors [18,19]. Thus, the independent association between ln(PIV) and malignancy in our study supports its potential inclusion in future multimarker preoperative algorithms. The relatively wide confidence intervals observed for ln(CA-125) and ln(PIV) may reflect scaling effects related to logarithmic transformation and the limited cohort size.

The prognostic findings require cautious interpretation. Previous studies have reported associations between systemic inflammatory indices and ovarian cancer outcomes [14,22,23,24]. However, the consistency and magnitude of these associations appear to vary according to disease stage, treatment setting, cohort composition, and statistical adjustment strategies. In the present cohort, preoperative PIV was not independently associated with either PFS or OS (log-rank *p* = 0.529 and *p* = 0.576, respectively).

The absence of prognostic significance in our cohort should be interpreted cautiously. The relatively limited number of survival events, moderate follow-up duration, and inclusion of only patients who achieved complete cytoreductive surgery may have reduced prognostic heterogeneity and limited the ability to detect modest associations between preoperative inflammatory burden and oncologic outcomes. In addition, potential time-dependent effects observed for certain clinicopathological covariates may have influenced the stability of the Cox regression estimates, particularly for overall survival analyses. This is also consistent with population-based evidence showing that survival in epithelial ovarian cancer is largely driven by established clinicopathological factors [25]. In the same direction, pooled evidence indicates that inflammation-based markers are prognostically relevant at the population level, but their apparent effect size varies according to stage, treatment setting, cut-off selection, adjustment status, and study quality [11,26]. Data from related gynecologic malignancies also suggest that PIV may show discriminatory value without consistently remaining independently prognostic in multivariable analysis [27].

The present study has several strengths. It evaluated PIV in a clinically relevant preoperative setting using routinely available hematologic parameters and included borderline ovarian tumors as a distinct category, allowing assessment across the full spectrum of ovarian tumor biology. In addition, the study incorporated multiple complementary analytical approaches, including ROC analysis, multivariable modeling, and survival assessment, within a surgically and pathologically confirmed cohort. Restriction of the malignant cohort to patients who underwent complete cytoreductive surgery also improved internal consistency for prognostic evaluation.

Several limitations should be acknowledged. First, the retrospective single-center design and restriction of the malignant cohort to patients who underwent complete cytoreductive surgery introduced a risk of selection bias and may limit the generalizability of the findings to broader ovarian cancer populations. Second, the relatively limited number of survival events and moderate follow-up duration may have reduced statistical power for prognostic analyses, particularly for overall survival. Third, inflammatory indices are dynamic biomarkers, and residual confounding cannot be fully excluded because they may be influenced by unmeasured host- and disease-related factors, including menopausal status, comorbidities, medications, occult inflammatory conditions, perioperative stress, and overall tumor burden despite the applied exclusion criteria. Fourth, because some patients were initially managed at external referral centers, complete confirmation of identical analytical platforms for all preoperative CA-125 and complete blood count measurements was not always possible, and potential inter-laboratory variability should therefore be considered. Finally, although internal validation and calibration analyses demonstrated favorable model performance, some degree of model optimism or overfitting cannot be completely excluded because external validation was unavailable. In addition, the optimal PIV cut-off remains uncertain, which may limit immediate clinical applicability.

## 5. Conclusions

PIV demonstrated the highest diagnostic performance among evaluated inflammatory indices. Its value was particularly evident in analyses including borderline ovarian tumors, a clinically important diagnostic gray zone. However, CA-125 retained the highest overall diagnostic accuracy, and the incremental contribution of PIV beyond CA-125 appeared modest. In this highly selected cohort of patients who underwent complete cytoreductive surgery, PIV did not demonstrate independent prognostic significance. Therefore, PIV should currently be considered a complementary inflammation-based biomarker within multimarker preoperative assessment strategies rather than a replacement for CA-125, pending validation in larger prospective multicenter cohorts representing broader ovarian cancer populations.

## Figures and Tables

**Table 1:** Baseline clinicopathological characteristics of patients according to tumor classification.

Variable	Benign (*n* = 139)	Borderline (*n* = 61)	Malignant (*n* = 116)	*p* Value
Age (years)	60.0 (49.5–68.0)	44.0 (32.0–50.0)	59.5 (50.0–68.0)	<0.001
BMI (kg/m^2^)	28.0 (25.6–31.0)	27.1 (24.9–28.8)	26.1 (23.9–28.4)	0.001
Tumor size (cm)	5.80 (4.30–6.80)	8.20 (7.20–9.70)	9.05 (7.83–10.55)	<0.001
CA-125 (U/mL)	18.90 (14.45–25.55)	43.80 (31.70–55.60)	182.30 (103.88–307.48)	<0.001
FIGO stage I–II	—	48 (78.7%)	31 (26.7%)	<0.001
FIGO stage III–IV	—	13 (21.3%)	85 (73.3%)	

Values are expressed as median (interquartile range) or *n* (%). BMI, body mass index; CA-125, cancer antigen 125; FIGO, International Federation of Gynecology and Obstetrics. *p* values represent comparisons among the three groups. FIGO staging was not applicable to benign tumors.

**Table 2:** Comparison of inflammation-based indices across benign, borderline, and malignant ovarian tumors.

Variable	Benign	Borderline	Malignant	*p* Value
PIV	264.13 (193.21–343.11)	399.39 (313.32–486.38)	524.28 (408.62–711.46)	<0.001
NLR	2.26 (1.77–2.73)	2.48 (2.22–2.84)	3.05 (2.56–3.96)	<0.001
PLR	129.44 (110.89–151.68)	154.32 (137.74–179.76)	190.91 (154.80–255.32)	<0.001
MLR	0.21 (0.17–0.24)	0.24 (0.20–0.27)	0.29 (0.22–0.36)	<0.001

Values are presented as median (interquartile range). PIV, pan-immune-inflammation value; NLR, neutrophil-to-lymphocyte ratio; PLR, platelet-to-lymphocyte ratio; MLR, monocyte-to-lymphocyte ratio. *p* values indicate overall comparisons among benign, borderline, and malignant ovarian tumor groups.

**Table 3:** Multivariable logistic regression analysis for predictors of malignancy.

Variable	OR (95% CI)	*p* Value
Age	1.10 (1.04–1.17)	0.001
Tumor size	1.65 (1.18–2.32)	0.004
ln(CA-125)	136.96 (25.02–749.76)	<0.001
ln(PIV)	63.81 (7.13–571.21)	<0.001

OR, odds ratio; CI, confidence interval; CA-125, cancer antigen 125; PIV, pan-immune-inflammation value; ln, natural logarithm. Variables shown were identified in the multivariable logistic regression model as independent predictors of malignancy.

**Table 4:** Clinicopathological characteristics of the prognostic malignant cohort according to preoperative PIV groups.

Variable	Overall (*n* = 116)	Low PIV (*n* = 58)	High PIV (*n* = 58)	*p* Value
Age, years	59.5 (50.0–68.0)	59.00 (51.00–67.00)	61.00 (49.00–69.50)	1.000
Tumor size, cm	9.05 (7.83–10.55)	8.75 (7.43–10.75)	9.30 (7.83–10.80)	0.525
CA-125, U/mL	182.30 (103.88–307.48)	178.96 (100.53–297.65)	183.20 (104.93–299.48)	0.830
PIV	524.28 (408.62–711.46)	406.26 (336.78–448.91)	714.41 (606.90–864.19)	<0.001
Histology: High-grade serous	71 (61.2%)	37 (63.8%)	34 (58.6%)	0.703
Histology: Clear cell	16 (13.8%)	5 (8.6%)	11 (19.0%)	0.177
Histology: Endometrioid	10 (8.6%)	5 (8.6%)	5 (8.6%)	1.000
Histology: Low-grade serous	10 (8.6%)	5 (8.6%)	5 (8.6%)	1.000
Histology: Mucinous carcinoma	9 (7.8%)	6 (10.3%)	3 (5.2%)	0.490
FIGO stage I–II	31 (26.7%)	18 (31.0%)	13 (22.4%)	0.402
FIGO stage III–IV	85 (73.3%)	40 (69.0%)	45 (77.6%)	0.402
Progression/recurrence	46 (39.7%)	25 (43.1%)	21 (36.2%)	0.569
Death	23 (19.8%)	13 (22.4%)	10 (17.2%)	0.642

PIV, pan-immune-inflammation value; FIGO, International Federation of Gynecology and Obstetrics; CA-125, cancer antigen 125.

**Table 5:** Cox proportional hazards regression analysis for PFS and OS.

Variable	PFS HR (95% CI)	*p* Value	OS HR (95% CI)	*p* Value
High vs. low PIV (univariable)	0.83 (0.46–1.48)	0.531	0.79 (0.35–1.81)	0.579
ln(PIV) (multivariable model)	0.93 (0.44–1.93)	0.835	1.60 (0.55–4.62)	0.389
Age (multivariable model)	0.99 (0.96–1.01)	0.251	0.99 (0.96–1.03)	0.743
FIGO stage (multivariable model)	1.67 (0.78–3.58)	0.185	1.23 (0.44–3.45)	0.692
Tumor size (multivariable model)	1.08 (0.91–1.28)	0.380	1.21 (0.95–1.53)	0.122
ln(CA-125) (multivariable model)	1.29 (0.80–2.06)	0.296	1.83 (0.90–3.69)	0.093

PFS, progression-free survival; OS, overall survival; HR, hazard ratio; CI, confidence interval; PIV, pan-immune-inflammation value; FIGO, International Federation of Gynecology and Obstetrics; CA-125, cancer antigen 125; ln, natural logarithm. Variables were analyzed using Cox proportional hazards regression.

## Data Availability

The data presented in this study are available from the corresponding author upon reasonable request. The data are not publicly available due to privacy and ethical restrictions.

## Abbreviations

The following abbreviations are used in this manuscript:

AUC	Area under the curve
BMI	Body mass index
CA-125	Cancer antigen 125
CI	Confidence interval
FIGO	International Federation of Gynecology and Obstetrics
HR	Hazard ratio
IQR	Interquartile range
MLR	Monocyte-to-lymphocyte ratio
NLR	Neutrophil-to-lymphocyte ratio
OR	Odds ratio
OS	Overall survival
PFS	Progression-free survival
PIV	Pan-immune-inflammation value
PLR	Platelet-to-lymphocyte ratio
ROC	Receiver operating characteristic
VIF	Variance inflation factor
